# Analysis of the Complete Mitochondrial Genome of the Bitter Gourd (*Momordica charantia*)

**DOI:** 10.3390/plants12081686

**Published:** 2023-04-17

**Authors:** Yu Niu, Ting Zhang, Muxi Chen, Guoju Chen, Zhaohua Liu, Renbo Yu, Xu Han, Kunhao Chen, Aizheng Huang, Changming Chen, Yan Yang

**Affiliations:** 1 Tropical Crops Genetic Resources Research Institute, Chinese Academy of Tropical Agricultural Sciences, Danzhou 571737, China; 2 College of Horticulture, South China Agricultural University, Guangzhou 510642, China; 3 Guangdong Helinong Biological Seeds Co., Ltd., Shantou 515800, China; 4 Guangdong Helinong Agricultural Research Institute Co., Ltd., Shantou 515800, China; 5 Institute of Agricultural Science Research of Jiangmen, Jiangmen 529060, China

**Keywords:** bitter gourd, mitochondrial genome, de novo assembly, phylogenetic analysis, RNA editing events, covariance analysis

## Abstract

Bitter gourd (*Momordica charantia* L.) is a significant vegetable. Although it has a special bitter taste, it is still popular with the public. The industrialization of bitter gourd could be hampered by a lack of genetic resources. The bitter gourd’s mitochondrial and chloroplast genomes have not been extensively studied. In the present study, the mitochondrial genome of bitter gourd was sequenced and assembled, and its substructure was investigated. The mitochondrial genome of bitter gourd is 331,440 bp with 24 unique core genes, 16 variable genes, 3 rRNAs, and 23 tRNAs. We identified 134 SSRs and 15 tandem repeats in the entire mitochondrial genome of bitter gourd. Moreover, 402 pairs of repeats with a length greater than or equal to 30 were observed in total. The longest palindromic repeat was 523 bp, and the longest forward repeat was 342 bp. We found 20 homologous DNA fragments in bitter gourd, and the summary insert length was 19,427 bp, accounting for 5.86% of the mitochondrial genome. We predicted a total of 447 potential RNA editing sites in 39 unique PCGs and also discovered that the ccmFN gene has been edited the most often, at 38 times. This study provides a basis for a better understanding and analysis of differences in the evolution and inheritance patterns of cucurbit mitochondrial genomes.

## 1. Introduction

Bitter gourd (*Momordica charantia* L.), also known as African cucumber, bitter melon, balsam pear, etc. [1], originated in tropical Africa and was domesticated in South Asia. It belongs to the botanical genus Momordica in the family Cucurbitaceae and is cultivated in the Middle East, Africa, India, and China as a tropical or semi-tropical plant [2]. Many physiologically active compounds and different vitamins can be found in bitter gourd. It is a great source of fiber and is abundant in minerals such as zinc, iron, magnesium, and calcium [3]. Bitter gourd, which has a very bitter flavor, is consumed as a vegetable in an immature state, whereas it is used as a condiment when it is ripe [4]. Bitter gourd has been widely used as a natural or folk cure for diabetes by the general public [5]. It was also used to alleviate symptoms of kidney stones, anthelmintics, gout, diabetes, skin conditions, and pneumonia [6,7,8]. Bitter gourd production is severely affected by biological adversities, such as powdery mildew, wilt disease, and anthrax, as well as abiotic stressors, such as flooding, drought, and chilling, which may cause lower quality and lower yields of bitter gourd. In the process of selecting and breeding new varieties with high resistance, due to the relatively weak genetic breeding research base of bitter gourd and the complexity of the genome, many valuable genes, particularly those for specific features originating from wild species, are difficult to incorporate into new types. At present, several studies have been reported on the genome and transcriptome of bitter gourd, laying the foundation for improving bitter gourd traits and yield.

The mitochondria are important organelles in eukaryotic cells, which are important sites for energy synthesis and conversion during the completion of life activities and provide energy security for various physiological activities of cells [9]. They have an independent mitochondrial genome, which usually demonstrates maternal inheritance [10]. The plant mitochondrial genome is characterized by: a large variation in genome size and structure; extremely conserved genes; very sparse gene distribution; large amounts of non-coding sequences; and large amounts of RNA editing events. In addition, the plant mitochondrial and chloroplast genomes are relatively independent of the cell nuclear genome in that they possess semi-autonomous heritable characteristics [11]. The conformation of plant mitochondrial genomes is diverse due to repeat sequences, and the assembled mitochondrial genomes may have cyclic, multi-loop, linear, and possibly multi-branched structures [12,13]. To date, 8814 chloroplast genomes and 1188 plastid genomes have been released to the NCBI database. However, according to NCBI, there are only 523 mitochondrial genomes in existence. (https://www.ncbi.nlm.nih.gov/genome/browse/#!/organelles/ (accessed on 20 October 2022)). It has been found that organelle genes in Cucurbitaceae crops are not only associated with important cellular metabolic pathways, such as photosynthesis and respiration, but also with important traits, such as cold resistance and sex differentiation [14,15,16]. In addition, there are significant mitochondrial genomic differences between different Cucurbitaceae crops. Therefore, the study of the mitochondrial genome of bitter gourd can help to investigate the evolutionary origin relationship of Cucurbitaceae crops and the role of mitochondrial genes in bitter gourd resistance. Due to the complex structure of plant mitochondrial genomes, research on these genomes has lagged behind that on chloroplast and plastid genomes. Currently, the mitochondrial genomes of cucurbits, such as cucumber [17] and watermelon and zucchini [18], have been sequenced. The whole genome of bitter gourd has been sequenced [19,20,21], while the mitochondrial genomes of bitter gourd have not yet been revealed.

In this research, we sequenced and assembled the mitochondrial genome of the bitter gourd. The characteristics of the genome sequence and differences between mitochondrial genomes of closely related species were analyzed, such as GC content, codon preference, repetitive sequence analysis, phylogenetic analysis, RNA editing events, sequence migration analysis, etc. It was anticipated that these would serve as a foundation for a deeper comprehension and investigation of the variations in the evolution and inheritance patterns of cucurbit mitochondrial genomes.

## 2. Results

### 2.1. Mitochondrial Genome Assembly and Annotation of Bitter Gourd

**Figure 1:**
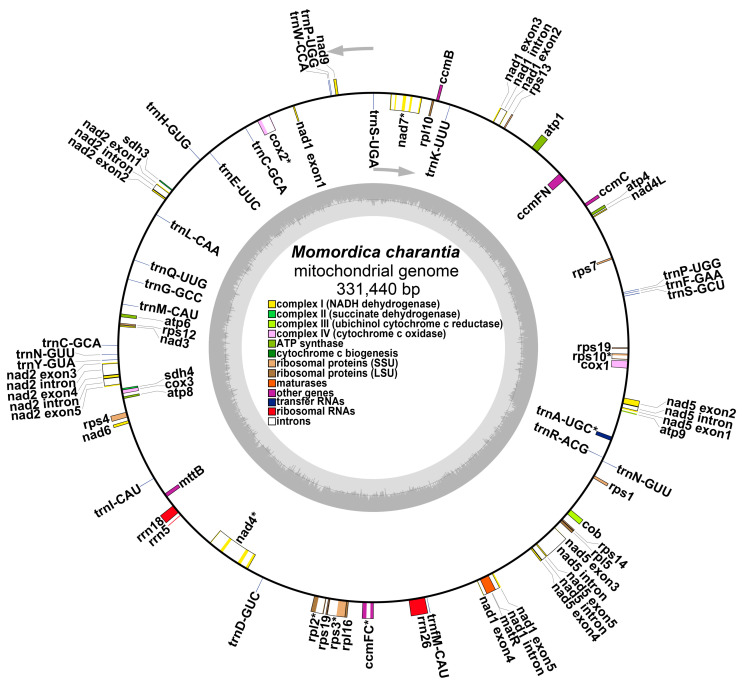
Genome circle map representing the mitochondrial genome of *Momordica charantia*. The colored squares inside and outside the circle represent various mitochondrial genes. Genes belonging to the same function are represented by the same color. Genes nad1, nad2, nad5, etc. are represented in the form of multiple exons and introns. * Represents non-trans-sheared intron genes. The arrows represent the forward and reverse chains.

After excluding duplicated regions in the Pacbio data, a hybrid assembly model was adopted, and its mitochondrial genome was temporarily presented as a molecular circle with 331,440 bp (Figure 1) and a GC content of 45.60%. The graphical mitochondrial genome assembled by GetOrganelle software was further processed using bwa software to obtain a sketch of the bitter gourd mitochondrial genome (Figure S1).

We annotated the mitochondrial genome of bitter gourd, and the classification of genes is shown in Table 1. The mitochondrial genome of bitter gourd is available in GenBank (https://www.ncbi.nlm.nih.gov/ (accessed on 10 March 2023)) with accession number OQ603604. The bitter gourd mitochondrial genome contains 24 unique core genes and 16 variable genes. The core genes include five ATP synthase genes, nine NADH dehydrogenase genes, four cytochrome c biogenesis genes, a ubiquinol cytochrome c reductase (cob), three cytochrome C oxidase genes (cox1, cox2, and cox3), a transport membrane protein (mttB), and a maturase (matR). The variable genes consist of four large ribosomal protein subunits, ten small ribosomal protein subunits, and two succinate dehydrogenase genes (sdh3 and sdh4). In all, 3 rRNAs and 23 tRNAs were annotated in the bitter gourd mitochondrial genome, with 3 tRNA genes that were double-copy genes, including trnC-GCA, trnN-GUU, and trnP-UGG. (Table 1).

### 2.2. The Structure and Codon Preference of the Mitochondrial Genome 

**Figure 2:**
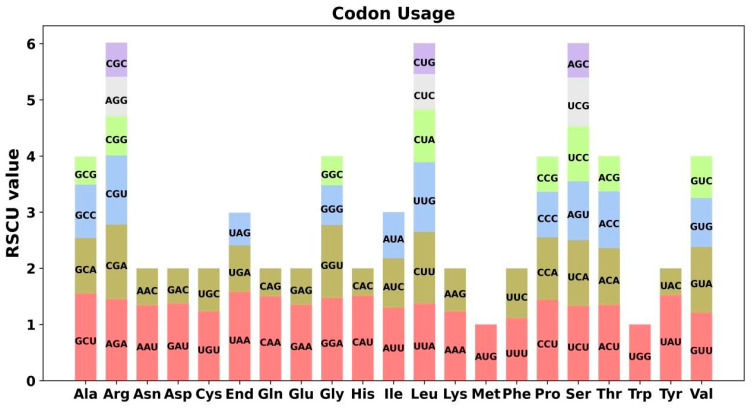
The codon preference analysis of the mitochondrial genome. The rectangles of different colors represent the RSCU values of different codons encoding the same amino acid.

Codon preference analysis was performed on 39 unique PCGs of *Momordica charantia* mitochondria, and codon usage of individual amino acids is shown in Table S1. Amino acids were thought to employ codons with relative synonymous codon use (RSCU) higher than 1 preferentially. As shown in Figure 2, except for the start codons AUG and tryptophan (UGG), both of which have RSCU values of 1, codon usage preference for mitochondrial PCGs is very widespread. For example, the termination codon has a high preference for the use of UAA, with the highest RSCU value of 1.58 among mitochondrial PCGs. Secondly, alanine (Ala) prefers the use of GCU, with an RSCU value of 1.55. It is worth noting that phenylalanine (Phe) has a maximum RSCU value of less than 1.2 and does not have a strong preference for codon usage.

### 2.3. Repeat Elements and DNA Transfer Analysis

**Figure 3:**
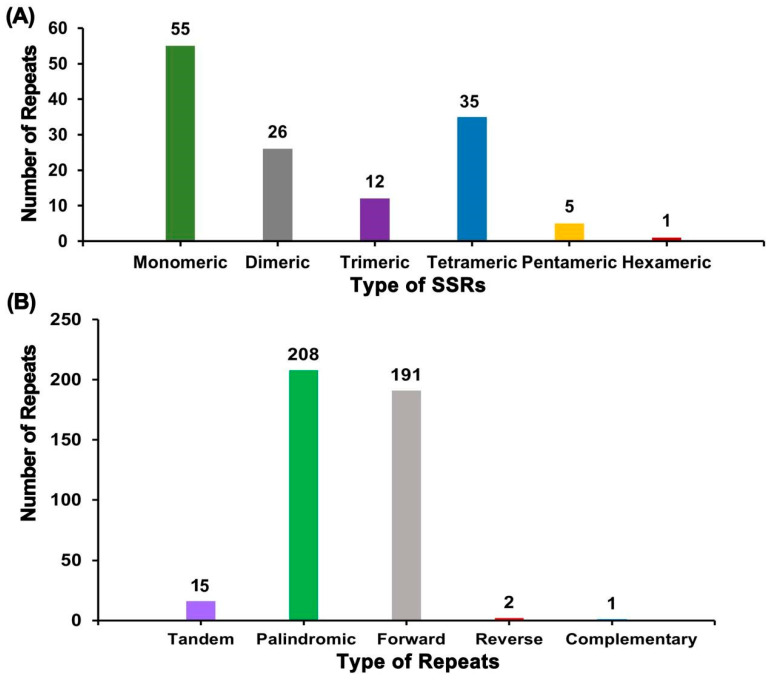
Repeat sequences analysis. (**A**) The horizontal coordinates indicate the type of SSR, and the vertical coordinates indicate the number of repeats. (**B**) The horizontal coordinates indicate the type of repetitive sequence, and the vertical coordinates indicate the number of repeats.

Because of the high polymorphism and codominant inheritance, microsatellites (simple sequence repeats [SSRs]) are frequently utilized for molecular marker design [22]. The Misa web server (https://webblast.ipk-gatersleben.de/misa/ (accessed on 15 September 2022) was used to gain SSRs in the mitochondrial genome of bitter gourd (Table S2, Figure 3A), and identified 134 SSRs. Among them, monomeric and dimeric SSRs accounted for 60.45% of the total SSRs. Thymine (T) monomeric repeats accounted for 54.55% (30) of the 55 monomeric SSRs, and TA repeats were the most common type of dimeric SSRs, accounting for 34.62% of dimeric SSRs. In the mitochondrial genome, there was only 1 hexameric SSR. We also detected tandem repeats and dispersed repeats in the mitochondrial genomes of bitter gourd (Tables S3 and S4, Figure 3B). Tandem repeats, also known as satellite DNA, are core repeat units of about 7–200 bases that are repeated in tandem multiple times. They are widely found in eukaryotic and prokaryotic genomes. There are 15 tandem repeats in the mitochondrial genome with a greater than 79% match and a length between 12 and 69 bp (Figure 3B). The mitochondrial genome was examined for scattered repeats. A total of 402 pairs of repeats with a length greater than or equal to 30 bp were found, including 208 pairs of palindromic repeats, 191 pairs of forward repeats, 2 pairs of reverse repeats, and 1 pair of complementary repeats. The longest palindromic repeat was 523 bp, while the longest forward repeat was 342 bp.

**Figure 4:**
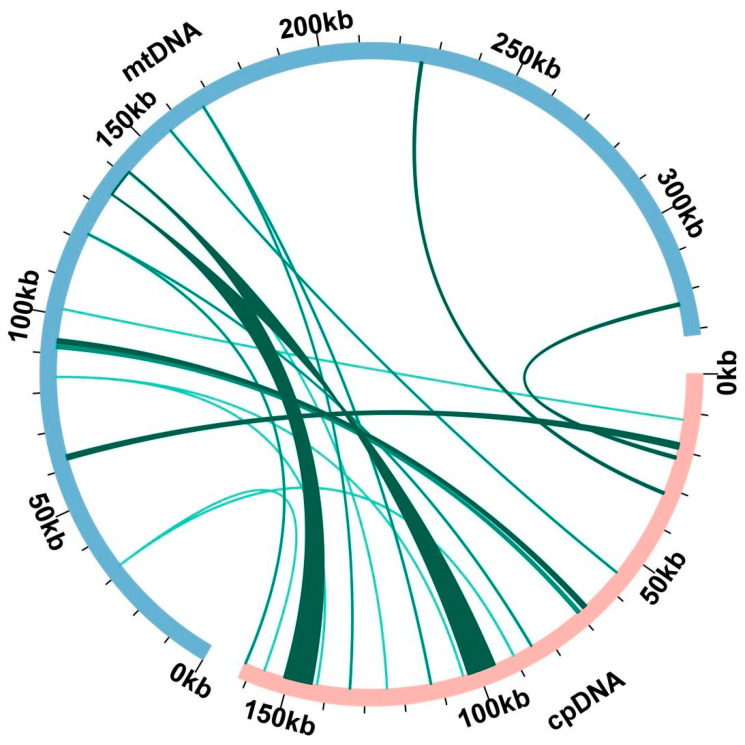
Homological sequences between mitochondrial and chloroplast genomes. The blue circular segment represents the mitochondrial genome, the pink circular segment represents the chloroplast genome, and the green line represents the homologous fragment.

Some chloroplast fragments were incorporated into the mitochondrial DNA throughout mitochondrial evolution, and the length of migrated fragments and sequence similarity vary among different species. Based on the sequence similarity analysis, we found 20 homologous DNA fragments in bitter gourd (Table S5, Figure 4). The total insert length was 19,427 bp, accounting for 5.86% of the mitochondrial genome. Fragment 1 and fragment 2 are the longest, with a length of 7382 bp. Annotation of these homologous sequences made it possible to identify 14 complete genes out of 20 homologous fragments, including 7 PCGs (petG, psbE, psbF, psbL, psbJ, rps7, ndhB) and 7 tRNA genes (trnHGUG, trnI-CAU, trnL-CAA, trnM-CAU, trnN-GUU, trnP-UGG, trnW-CCA).

### 2.4. Phylogenetic Analysis and RNA Editing Events

**Figure 5:**
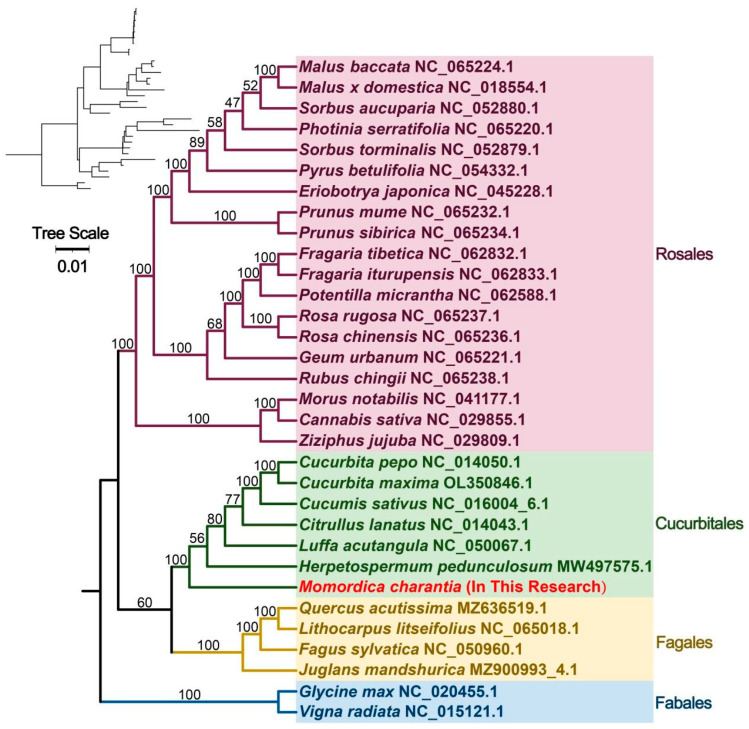
Phylogenetic relationships of bitter gourd.

To further explore the evolutionary relationships of mitochondria in bitter gourd, 32 mitochondrial genomes from four angiosperm orders were selected for phylogenetic analysis, including 19 species of Rosales, 7 species of Cucurbitales, 4 species of Fagales, and 2 species of Fabales. There is a lot of structural variety between these species. Therefore, we adopted a shared, conserved PCG tree construction approach. Phylogenetic analysis was performed on 21 PCGs (atp1, atp4, atp6, atp8, ccmB, ccmC, ccmFc, ccmFn, cox1, cox3, nad1, nad2, nad3, nad4, nad6, nad7, nad9, rpl16, rps3, rps4, and sdh4) (Figure 5). According to our analysis, the topological structure of mitochondrial DNA-based phylogeny coincides with the latest APG classification (Angiosperm Phylogeny Group). Bitter gourd, belonging to the Cucurbitaceae family, is closely related to *Herpetospermum pedunculosum* (Polyphemus).

**Figure 6:**
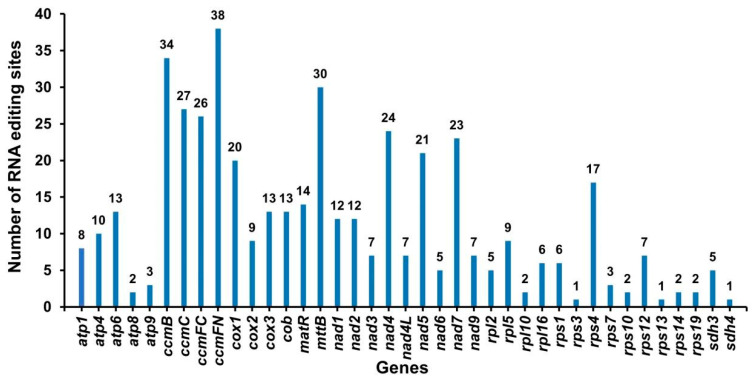
Number of RNA editing sites in all the PCGs.

RNA editing events were identified for 39 unique PCGs based on online website predictions. The standard was set to a threshold value of 0.001, and under this standard, there were 447 potential RNA editing sites distributed among all PCGs (Table S6, Figure 6). Finally, we only found C → U editing in this mitochondrial genome. The number of RNA editing sites in different PCGs ranges from 1 to 38. The ccmFN gene has the most RNA editing sites (38 sites, 8.5%), followed by the ccmB gene, which has 34 RNA editing sites. The rps3, rps13, and sdh4 genes have the lowest number of RNA editing events, with only one site.

### 2.5. Covariance Analysis

**Figure 7:**
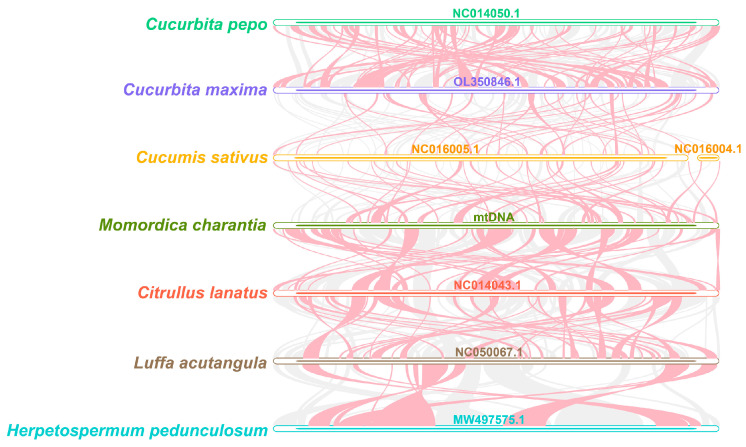
Covariance analysis. The red curved areas indicate regions where inversions occur, and the gray areas indicate regions with good homology.

*Cucurbita pepo*, *Cucurbita maxima*, *Cucumis sativus*, *Momordica charantia*, *Citrullus lanatus*, *Luffa acutangula*, and *Herpetospermum pedunculosum* were selected for covariance analysis. As shown in Figure 7, a large number of homologous collinear blocks were detected between the *Momordica charantia* mitochondrial genome and the other six Cucurbitaceae species, but the length of these collinear blocks was short. In addition, the discovery of some gaps illustrates that these sequences are unique to the species and have no homology with the rest of the species. The results suggest that the collinear blocks between the different mitochondrial genomes of Cucurbitaceae are not in the same order, and the *Momordica charantia* mitochondrial genome has undergone a lot of genomic rearrangement with close species. The short length of the collinear blocks indicates that the mitochondrial genome sequences of the seven species of Cucurbitaceae are extremely unconservative and undergo extremely frequent genomic rearrangements.

## 3. Discussion

The mitochondrial genome structures of all currently sequenced cucurbits are cyclic, while the mitochondrial genome structure varied significantly among different Cucurbitaceae crops. For example, the cucumber mitochondrial genome contains a large main loop and two small subloop structures with a size of about 1685 kb [17]. In contrast, single loops of 379 kb and 983 kb were found in both watermelon and zucchini, respectively [18]. In this study, we sequenced and assembled the bitter gourd mitochondrial genome as a molecular circle with a size of approximately 331 kb. The mitochondrial genome of muskmelon is the largest in the Cucurbitaceae family [23], with sizes 2, 3, and 7 times larger than cucumber, zucchini, and bitter gourd, respectively.

Horizontal gene transfer (HGT) is the process of transferring genetic material across cells or organelles. It is very widespread in different organelle genomes (mitochondria and chloroplasts), and nuclear genomes are also rich in genetic material exchange with organelle genomes. In this study, based on sequence similarity analysis, we found 20 homologous DNA fragments in bitter gourd. The total insert length accounted for 5.86% of the mitochondrial genome. A few chloroplast sequences were discovered in the mitochondrial genomes of cucurbit crops, with the most in zucchini, followed by watermelon, cucumber, and muskmelon. In addition, studies have shown that HGT also exists between plants and prokaryotes. For example, sequences similar to those of proteus bacillus and mitochondrial virus were found in the cucumber mitochondrial genome [17], while there are no similar reports in other Cucurbitaceae crops.

RNA editing is a very common phenomenon in the mitochondrial genomes of higher plants, and the total number of RNA edits in Arabidopsis, rice, and oilseed rape all exceeded 400 [24,25,26]. Almost all of the transcription products of mitochondrial PCGs are subjected to varying degrees of RNA editing but rarely occur in rRNA, tRNA, and introns [24]. The total number of RNA editing sites in watermelon and zucchini were 463 and 444, respectively, both of which were C → U transitions. In our research, we found 447 potential RNA editing sites in the bitter gourd mitochondrial genome, both of which were also C → U editing. The RNA editing time for each gene is quite different in Cucurbitaceae crops. Ribosomal protein genes (rpl2, rps1, and rps7) have fewer RNA editing events than other genes, while genes such as mttb, ccmB, and ccmFn have a higher number of RNA editing events, which is highly consistent in Cucurbitaceae crops.

There are many repetitive sequences distributed in the mitochondrial genome of Cucurbitaceae crops, and their length and conformation are highly diverse. In this study, a total of 402 pairs of repeats were observed, including 208 pairs of palindromic repeats, 191 pairs of forward repeats, 2 pairs of reverse repeats, and 1 pair of complementary repeats. The Muskmelon mitochondrial genome contains almost half of the repetitive sequences [23]; the mitochondrial genomes of cucumber and zucchini contain 35.9% and 37.7% repetitive sequences, respectively [17,18], both of which represent more than one third of the total sequences, whereas the repetitive sequence in the watermelon mitochondrial genome is only 10.0% of the whole genome. The recombination of repetitive sequences in the mitochondrial genome has a great impact on the genome size, gene arrangement, and evolution of the mitochondrial genome of Cucurbitaceae crops and may also lead to plant phenotypic mutations. For example, mosaic phenotypic mutant lines of cucumber obtained by self-crossing for several generations after tissue culture screening of mutants may be related to the duplication or recombination of mitochondrial genome sequences [27,28,29]. In other higher plants, frequent recombination of repetitive sequences also leads to cytoplasmic male sterility, such as in maize [30] and beets [31]. It can also change the position of the promoter to affect the gene expression pattern [32]. Dissecting the mitochondrial genome of bitter gourd may provide a theoretical basis for CMS breeding in bitter gourd.

## 4. Materials and Methods

### 4.1. Plant Materials, DNA Extraction, and Sequencing

We obtained the fresh bitter gourd plant leaves from the Danzhou Team 5 Test Base of the Tropical Crop Variety Resources Research Institute. Then the leaves were cleaned with DEPC water and kept in a freezer at −80 °C. The DNA of bitter gourd was extracted by a DNA plant extraction kit (Tiangen, Beijing, China).

We employed a hybrid assembly method to assemble the bitter gourd mitochondrial genome. Specifically, the short-paired reads and long-paired reads were sequenced, respectively, using Illumina HiSeq X Ten (Illumina, Inc.; San Diego, CA, USA) and PacBio RS II. Both Illumina paired-end short reads and PacBio long reads were acquired. Moreover, the Illumina raw reads have been modified to remove poor-quality bases [33].

### 4.2. Genome Assembly and Annotation

We used Illumina second-generation sequencing data and PacBio third-generation sequencing data to assemble the bitter gourd mitochondrial genome using a hybrid assembly strategy. The GetOrganelle software (Kunming Institute of Botany, Chinese Academy of Sciences, Kunming, China; default parameters: v1.7.5) [34] was used to perform graphical plant mitochondrial assembly on second-generation DNA sequencing data to obtain a graphical plant mitochondrial genome. The graph-based mitochondrial genome was visualized by the Bandage program (parameters: v0.8.1) [35], and the chloroplast and single stretches of the nuclear genome were manually excised. The PacBio data were then compared to the graphical mitochondrial genome using the bwa software (Toulouse, France; parameters: v0.7.17) [36]. The PacBio data obtained were used to solve regions of repetitive sequence in the graphed mitochondrial genome. 

The *Arabidopsis thaliana* genome was selected as the reference genome (accession number: NC_037304.1), and the protein-coding genes of the mitochondrial genome were identified. Geseq software (Potsdam-Golm, Germany; parameters: v2.03) [37] and CPGAVAS2 [38] were used to annotate the mitochondrial genome. The tRNAs and rRNAs of the mitochondrial genome were annotated with the tRNAscan-SE software (Santa Cruz, CA, USA; parameters: v2.0.12) [39] and BLASTN software (National Center for Biotechnology Information, Bethesda, MD, USA; parameters: v2.13.0) [40], respectively. The annotation of the mitochondrial genome was manually corrected with Apollo software (parameters: v1.11.8) [41].

### 4.3. Structural Analysis and Codon Preference Analysis

To resolve the repetitive regions in the obtained graphical mitochondrial genome, the long reads were compared with repetitive sequences using the bwa software to determine whether the repetitive regions are spanned by long reads and thus derive the most likely mitochondrial genomic structure.

The protein-coding sequences of the mitochondrial genome were extracted using Phylosuite software (Wuhan, Hubei, China; parameters: v1.1.16) [42]. Mega 7.0 software [43] was used to perform codon preference analysis, and RSCU values were calculated.

### 4.4. Repeat and Homologous DNA Analysis

Microsatellite repeats, tandem repeats, and dispersed repeats were identified by the MISA web server (https://webblast.ipk-gatersleben.de/misa/ (accessed on 20 September 2022)) (parameters: v2.1) [44], the TRF web server (https://tandem.bu.edu/trf/trf.unix.help.html (accessed on 20 September 2022)) (parameters: v4.09) [45], and the REPuter web server (https://bibiserv.cebitec.uni-bielefeld.de/reputer/ (accessed on 22 September 2022)) [46], respectively. Excel (2021) software was used to visualize the results. BLASTN revealed homologous DNA segments with an e-value of 1e-6 between the chloroplast genome and the mitochondrial genome. The findings were visualized by Circos package (Michael Smith Genome Sciences Centre, BC Cancer Agency, Vancouver, BC, Canada; parameters: v0.69-9A) [47].

### 4.5. Phylogenetic Analysis and RNA Editing Site Prediction

Thirty-two mitochondrial genomes from four angiosperm orders were chosen for phylogenetic analysis, with two Fabales mitochondrial genomes set as the outgroup. BLASTN was used to filter and retrieve mitochondrial genome common genes, which were then concatenated with Phylosuite. Multiple sequence alignment was performed using MAFFT (parameters: v7.505) [48]. A phylogenetic analysis was then performed by the IQ-TREE software using the method of maximum-likelihood (parameters: v1.6.12) [49]. Finally, the phylogenetic tree was visualized using iTOL (https://itol.embl.de/ (accessed on 25 September 2022)) (parameters: v6) [50]. We selected 25 species (Table S7) in the database for RNA prediction on the PREPACT3 website (http://www.prepact.de/ (accessed on 5 October 2022)) (parameters: v3.12.0) [51].

### 4.6. Covariance Analysis

*Cucurbita pepo*, *Cucurbita maxima*, *Cucumis sativus*, *Momordica charantia*, *Citrullus lanatus*, *Luffa acutangula*, and *Herpetospermum pedunculosum* were selected for covariance analysis. Based on BLASTN results for two-by-two comparisons of these seven mitochondrial genomes, homologous sequences longer than 500 bp were retained as conserved co-linear blocks for plotting the Multiple Synteny Plot.

## 5. Conclusions

In this study, we successfully sequenced and assembled the mitochondrial genome of bitter gourd, which made it possible for us to make a comprehensive comparison between the organelle genomes of bitter gourd, thus offering a broader perspective for the study of gene transfer between mitochondria and plastid. At the same time, the results of our covariance analysis provide information on the mitochondrial genomes of Cucurbitaceae crops, which may facilitate genomic structure investigations and an analysis of differences in the evolution and inheritance patterns of cucurbit mitochondrial genomes. 

## Figures and Tables

**Table 1:** Gene composition in the mitogenome of bitter gourd.

Group of Genes		Name of Genes
Core genes	ATP synthase	atp1, atp4, atp6, atp8, atp9
	NADH dehydrogenase	nad1, nad2, nad3, nad4, nad4L, nad5, nad6, nad7, nad9
	Cytochrome c biogenesis	ccmB, ccmC, ccmFC, ccmFN
	Ubiquinol cytochrome c reductase	cob
	Cytochrome c oxidase	cox1, cox2, cox3
	Maturases	matR
	Transport membrane protein	mttB
Variable genes	Large subunit of ribosome	rpl2, rpl5, rpl10, rpl16
	Small subunit of ribosome	rps1, rps3, rps4, rps7, rps10, rps12, rps13, rps14, rps19 (×2)
	Succinate dehydrogenase	sdh3, sdh4
rRNA genes	Ribosome RNA	rrn5, rrn18, rrn26
tRNA genes	Transfer RNA	trnA-UGC, trnC-GCA (×2), trnD-GUC, trnEUUC, trnF-GAA, trnfM-CAU, trnG-GCC, trnHGUG, trnI-CAU, trnK-UUU, trnL-CAA, trnMCAU, trnN-GUU (×2), trnP-UGG (×2), trnQUUG, trnR-ACG, trnS-GCU, trnS-UGA, trnWCCA, trnY-GU

## Data Availability

The mitochondrial genome of bitter gourd is available in GenBank (https://www.ncbi.nlm.nih.gov/) with accession number OQ603604.

## Supplementary Materials

The following are available online at https://www.mdpi.com/article/10.3390/plants12081686/s1, Figure S1: Sketch of the mitochondrial genome of bitter gourd, Table S1: RSCU for each amino acid pair of codons in the mitochondrial genome, Table S2: SSRs in the mitogenome, Table S3: tandem repeats in the mitogenome, Table S4: Dispersed repeats in the mitogenome, Table S5: The homologous DNA fragment in the bitter gourd mitochondrial genome, Table S6: The RNA editing events prediction in bitter gourd, Table S7: Species information for RNA editing event reference.
