# Supplementary material for: Analysis of the Complete Mitochondrial Genome of the Bitter Gourd (Momordica charantia)
Source: Plants (Basel). 2023 Apr 17;12(8):1686. doi: 10.3390/plants12081686 (PMC10143269; doi:10.3390/plants12081686)
Supplement: Supplementary file 1 [file plants-12-01686-s001.zip › plants-2189405-supplementary.pdf]

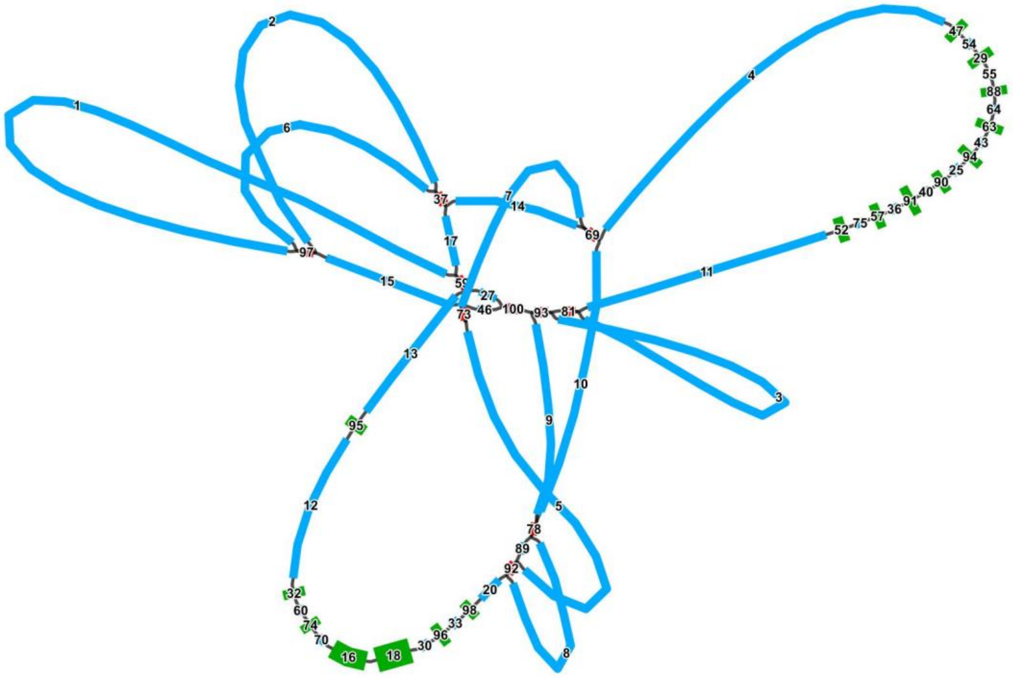

Figure S1. Sketch of the mitochondrial genome of bitter gourd. The red nodes are the predicted repeat regions, the green nodes are the predicted chloroplast genome migration to the mitochondrial genome, and the blue represents a single copy of the loop contig.

Table S1. RSCU for each amino acid pair of codons in the mitochondrial genome.

Amino	Codon 1 RSCU	Codon 2 RSCU	Codon 3 RSCU	Codon 4 RSCU	Codon 5 RSCU	Codon 6 RSCU
Ala	GCU	GCA	GCC	GCG		
	1.55	0.99	0.95	0.5		
Arg	AGA	CGA	CGU	CGG	AGG	CGC
	1.45	1.33	1.23	0.7	0.7	0.61
Asn	AAU	AAC				
	1.34	0.66				
Asp	GAU	GAC				
	1.37	0.63				
Cys	UGU	UGC				
	1.23	0.77				
End	UAA	UGA	UAG			
	1.58	0.83	0.58			
Gln	CAA	CAG				
	1.5	0.5				
Glu	GAA	GAG				
	1.35	0.65				
Gly	GGA	GGU	GGG	GGC		
	1.47	1.3	0.71	0.52		
His	CAU	CAC				
	1.51	0.49				
Ile	AUU	AUC	AUA			
	1.3	0.88	0.82			
Leu	UUA	CUU	UUG	CUA	CUC	CUG
	1.37	1.28	1.24	0.94	0.63	0.55
Lys	AAA	AAG				
	1.23	0.77				
Met	AUG					
	1					
Phe	UUU	UUC				
	1.11	0.89				
Pro	CCU	CCA	CCC	CCG		
	1.44	1.11	0.81	0.63		
Ser	UCU	UCA	AGU	UCC	UCG	AGC
	1.33	1.17	1.05	0.98	0.87	0.61
Thr	ACU	ACA	ACC	ACG		
	1.35	1.01	1.01	0.63		
Trp	UGG					
	1					
Tyr	UAU	UAC				
	1.52	0.48				
Val	GUU	GUA	GUG	GUC		
	1.21	1.17	0.87	0.75		

Table S2 SSRs in the mitogenome

Mitochondrial genome	SSR nr.	SSR type	SSR	size	start	end
Momordica charantia	1	p3	(AGA)4	12	539	550
Momordica charantia	2	p1	(A)11	11	3368	3378
Momordica charantia	3	p4	(TTCT)3	12	7336	7347
Momordica charantia	4	p4	(GCCA)3	12	12795	12806
Momordica charantia	5	p2	(TA)5	10	13618	13627
Momordica charantia	6	p4	(TTCT)3	12	16284	16295
Momordica charantia	7	p2	(TC)5	10	18249	18258
Momordica charantia	8	p3	(CTC)4	12	19694	19705
Momordica charantia	9	p1	(T)11	11	19987	19997
Momordica charantia	10	p4	(CAAA)3	12	35025	35036
Momordica charantia	11	p4	(CTTA)3	12	37235	37246
Momordica charantia	12	p4	(AAAG)3	12	39583	39594
Momordica charantia	13	p4	(AGAA)3	12	41729	41740
Momordica charantia	14	p6	(TCTCAA)3	18	43746	43763
Momordica charantia	15	p2	(TA)5	10	48336	48345
Momordica charantia	16	p4	(ATGG)3	12	52838	52849
Momordica charantia	17	p1	(T)13	13	53406	53418
Momordica charantia	18	p2	(AG)5	10	53922	53931
Momordica charantia	19	p1	(A)10	10	58013	58022
Momordica charantia	20	p4	(CTAG)3	12	58744	58755
Momordica charantia	21	p1	(A)11	11	61004	61014
Momordica charantia	22	p4	(CAAT)3	12	61958	61969
Momordica charantia	23	p4	(AAAG)3	12	64719	64730
Momordica charantia	24	p3	(TTC)5	15	64954	64968
Momordica charantia	25	p5	(GAAGG)3	15	67183	67197
Momordica charantia	26	p4	(ACTC)3	12	68511	68522
Momordica charantia	27	p1	(A)11	11	69951	69961
Momordica charantia	28	p1	(C)10	10	75090	75099
Momordica charantia	29	p5	(ACTAA)3	15	76906	76920
Momordica charantia	30	p1	(A)11	11	77453	77463
Momordica charantia	31	p4	(GGTC)3	12	78852	78863
Momordica charantia	32	p4	(TTGG)3	12	88730	88741
Momordica charantia	33	p1	(A)12	12	89667	89678
Momordica charantia	34	p1	(A)10	10	91823	91832
Momordica charantia	35	p2	(TA)5	10	99205	99214
Momordica charantia	36	p4	(CCTT)3	12	100632	100643
Momordica charantia	37	p2	(AT)8	16	100715	100730
Momordica charantia	38	p2	(AT)8	16	100733	100748
Momordica charantia	39	p4	(GAGC)3	12	104983	104994
Momordica charantia	40	p3	(CAA)4	12	105576	105587
Momordica charantia	41	p1	(T)10	10	106105	106114

Momordica charantia	42	p1	(T)10	10	106480	106489
Momordica charantia	43	p1	(C)10	10	111382	111391
Momordica charantia	44	p3	(GCT)4	12	113433	113444
Momordica charantia	45	p2	(TC)5	10	114033	114042
Momordica charantia	46	p1	(C)10	10	118906	118915
Momordica charantia	47	p4	(AAAG)3	12	120068	120079
Momordica charantia	48	p4	(AAGA)3	12	121073	121084
Momordica charantia	49	p1	(A)11	11	121317	121327
Momordica charantia	50	p2	(TC)5	10	123182	123191
Momordica charantia	51	p1	(T)10	10	124759	124768
Momordica charantia	52	p2	(AG)5	10	128168	128177
Momordica charantia	53	p1	(A)10	10	129730	129739
Momordica charantia	54	p1	(T)13	13	130456	130468
Momordica charantia	55	p2	(TA)5	10	133278	133287
Momordica charantia	56	p2	(TA)6	12	142253	142264
Momordica charantia	57	p3	(AAG)4	12	143911	143922
Momordica charantia	58	p4	(ATGA)3	12	147784	147795
Momordica charantia	59	p4	(GAAT)3	12	153383	153394
Momordica charantia	60	p1	(T)10	10	154128	154137
Momordica charantia	61	p1	(T)10	10	156147	156156
Momordica charantia	62	p2	(CT)5	10	156406	156415
Momordica charantia	63	p1	(T)10	10	158565	158574
Momordica charantia	64	p4	(ATAA)3	12	158650	158661
Momordica charantia	65	p2	(TC)5	10	162346	162355
Momordica charantia	66	p4	(TTCA)3	12	167630	167641
Momordica charantia	67	p1	(T)10	10	168347	168356
Momordica charantia	68	p5	(TTAGT)3	15	169884	169898
Momordica charantia	69	p1	(T)10	10	170377	170386
Momordica charantia	70	p4	(TTTG)3	12	170515	170526
Momordica charantia	71	p1	(A)11	11	173634	173644
Momordica charantia	72	p1	(T)12	12	173719	173730
Momordica charantia	73	p2	(CT)6	12	173976	173987
Momordica charantia	74	p1	(T)10	10	176855	176864
Momordica charantia	75	p2	(AT)5	10	186175	186184
Momordica charantia	76	p4	(TGAA)3	12	187079	187090
Momordica charantia	77	p1	(A)12	12	188937	188948
Momordica charantia	78	p2	(CA)5	10	192292	192301
Momordica charantia	79	p2	(TA)6	12	195498	195509
Momordica charantia	80	p1	(T)10	10	195544	195553
Momordica charantia	81	p1	(A)12	12	196320	196331
Momordica charantia	82	p1	(T)10	10	197124	197133
Momordica charantia	83	p2	(TC)5	10	197485	197494
Momordica charantia	84	p1	(T)12	12	197512	197523
Momordica charantia	85	p2	(AG)5	10	197687	197696

Momordica charantia	86	p4	(ATTG)3	12	207133	207144
Momordica charantia	87	p1	(A)10	10	207741	207750
Momordica charantia	88	p1	(T)10	10	212522	212531
Momordica charantia	89	p1	(A)10	10	214328	214337
Momordica charantia	90	p4	(TTGC)3	12	217573	217584
Momordica charantia	91	p2	(TC)7	14	219966	219979
Momordica charantia	92	p1	(A)10	10	224069	224078
Momordica charantia	93	p1	(A)11	11	224161	224171
Momordica charantia	94	p4	(TTCT)3	12	224380	224391
Momordica charantia	95	p1	(T)11	11	224614	224624
Momordica charantia	96	p1	(T)11	11	225314	225324
Momordica charantia	97	p1	(T)10	10	225433	225442
Momordica charantia	98	p4	(AAAG)3	12	227834	227845
Momordica charantia	99	p1	(C)10	10	237801	237810
Momordica charantia	100	p1	(T)10	10	240227	240236
Momordica charantia	101	p1	(A)10	10	240242	240251
Momordica charantia	102	p4	(CATT)3	12	241588	241599
Momordica charantia	103	p1	(A)14	14	243027	243040
Momordica charantia	104	p4	(GTAT)3	12	243777	243788
Momordica charantia	105	p3	(GAC)4	12	247792	247803
Momordica charantia	106	p3	(CTT)4	12	249811	249822
Momordica charantia	107	p3	(TTC)4	12	252931	252942
Momordica charantia	108	p1	(A)11	11	253962	253972
Momordica charantia	109	p3	(TCT)4	12	262725	262736
Momordica charantia	110	p3	(TTC)4	12	263509	263520
Momordica charantia	111	p2	(GA)5	10	268104	268113
Momordica charantia	112	p2	(TA)6	12	269477	269488
Momordica charantia	113	p1	(T)10	10	270571	270580
Momordica charantia	114	p5	(ACTAG)3	15	273222	273236
Momordica charantia	115	p1	(T)11	11	274961	274971
Momordica charantia	116	p1	(T)10	10	282607	282616
Momordica charantia	117	p3	(GAT)4	12	283259	283270
Momordica charantia	118	p1	(T)10	10	286412	286421
Momordica charantia	119	p4	(CTGC)3	12	288682	288693
Momordica charantia	120	p2	(TA)7	14	289727	289740
Momordica charantia	121	p2	(TA)5	10	293880	293889
Momordica charantia	122	p4	(TCTT)3	12	298101	298112
Momordica charantia	123	p1	(T)11	11	299928	299938
Momordica charantia	124	p4	(CAGC)3	12	301065	301076
Momordica charantia	125	p1	(A)10	10	303781	303790
Momordica charantia	126	p4	(TACT)3	12	304714	304725
Momordica charantia	127	p1	(T)10	10	305332	305341
Momordica charantia	128	p1	(T)12	12	305805	305816
Momordica charantia	129	p5	(ATATG)3	15	306843	306857

Momordica charantia	130	p2	(CT)5	10	308000	308009
Momordica charantia	131	p1	(A)10	10	317188	317197
Momordica charantia	132	p1	(T)11	11	318690	318700
Momordica charantia	133	p4	(GCCG)3	12	321671	321682
Momordica charantia	134	p1	(T)10	10	330632	330641

Table S3 tandem repeats in the mitogenome

Indices	Period Size	Copy Number	Consensus Size	Percent Matches	Percent Indels	Score	A	C	G	T	Entropy (0-2)
23371--23412	18	2.3	18	95	0	77	35	16	16	30	1.92
54125--54150	12	2.2	12	100	0	52	7	30	0	61	1.24
100701--100752	18	2.9	18	94	0	90	50	0	0	50	1
100701--100757	22	2.6	22	85	0	86	49	0	0	50	1
126130--126161	16	2	16	100	0	64	25	31	0	43	1.55
185267--185299	18	1.9	18	88	11	52	36	15	21	27	1.93
193191--193246	26	2.2	26	84	9	75	19	12	21	46	1.83
243201--243247	24	2	24	86	0	73	25	25	23	25	2
248610--248649	19	2	20	80	4	52	30	10	27	32	1.89
257939--257980	18	2.3	18	80	19	52	19	19	26	35	1.95
264479--264644	69	2.4	69	94	0	297	36	13	18	32	1.89
287005--287036	14	2.3	14	83	0	50	18	21	15	43	1.87
288288--288329	18	2.3	18	79	0	56	21	30	4	42	1.73
311966--311994	15	1.9	15	92	0	51	27	6	27	37	1.82
314266--314293	14	2	14	100	0	56	28	7	14	50	1.69

Table S4 Dispersed repeats in the mitogenome

The repeat length of the first part	The starting site of the first part	Matching direction	The repeat length of the second part	The starting site of the second part	Interval distance of repeats	E-value
523	177518	P	523	235968	0	0.00E+00
342	121362	F	342	240502	0	3.85E-196
331	239215	P	331	331109	0	1.61E-189
267	76779	P	267	169757	0	5.49E-151
255	89788	P	255	107853	-1	7.05E-141
243	58916	F	243	274270	0	1.55E-136
225	89818	P	225	107853	0	1.06E-125
180	240469	P	180	273622	0	1.32E-98
168	107527	P	168	302279	-2	2.79E-86
158	107537	P	158	302279	-1	1.10E-82
150	107545	P	150	302279	0	1.52E-80
147	121362	P	147	273622	0	9.71E-79
125	53746	F	125	200938	0	1.71E-65
128	291433	F	128	318238	-2	1.95E-62
115	298338	P	115	321789	-1	6.18E-57
102	89883	F	102	289836	0	1.20E-51
102	107911	P	102	289836	0	1.20E-51
101	89953	P	101	159464	0	4.81E-51
99	255829	F	99	317976	0	7.69E-50
98	37361	F	98	217732	-1	9.04E-47
90	107853	F	90	159475	0	2.02E-44
90	298363	P	90	321789	0	2.02E-44
79	36647	P	79	291623	0	8.46E-38
76	57556	F	76	121509	0	5.41E-36
76	57556	F	76	240649	0	5.41E-36
76	291485	F	76	318290	0	5.41E-36
82	37450	P	82	318047	-2	3.95E-35
81	54937	F	81	105764	-2	1.54E-34
84	264491	F	84	264560	-3	2.12E-34
73	80084	F	73	313960	0	3.46E-34
80	167102	F	80	307125	-3	4.69E-32
68	45439	P	68	177731	0	3.55E-31
68	45439	F	68	236210	0	3.55E-31
65	157517	P	65	203009	0	2.27E-29
67	157416	F	67	178120	-1	2.85E-28
65	37467	P	65	318047	-1	4.43E-27
64	19319	P	64	273937	-1	1.74E-26
64	55017	F	64	105841	-1	1.74E-26

60	146635	F	60	149690	0	2.32E-26
59	80145	P	59	314024	0	9.30E-26
62	240109	F	62	319351	-1	2.70E-25
56	59111	P	56	160325	0	5.95E-24
56	67882	P	56	263660	0	5.95E-24
63	216897	F	63	237743	-2	6.38E-24
66	54227	F	66	271223	-3	7.01E-24
65	57463	F	65	157417	-3	2.68E-23
60	74070	P	60	105401	-2	3.70E-22
60	239217	F	60	282544	-2	3.70E-22
60	282544	P	60	331378	-2	3.70E-22
53	54965	F	53	105792	0	3.81E-22
63	19308	P	63	273949	-3	3.89E-22
63	54876	F	63	105701	-3	3.89E-22
52	57468	F	52	178126	0	1.52E-21
52	60590	P	52	158584	0	1.52E-21
55	123650	F	55	240492	-1	3.93E-21
55	123650	P	55	273724	-1	3.93E-21
54	42482	F	54	158583	-1	1.54E-20
60	59021	F	60	318530	-3	2.15E-20
60	274375	F	60	318530	-3	2.15E-20
50	100706	P	50	100706	0	2.44E-20
59	46106	F	59	302207	-3	8.16E-20
49	57047	F	49	132733	0	9.75E-20
52	42483	P	52	60590	-1	2.38E-19
48	160333	P	48	274465	0	3.90E-19
50	74080	P	50	105401	-1	3.66E-18
50	239227	F	50	282554	-1	3.66E-18
50	282554	P	50	331378	-1	3.66E-18
46	37486	P	46	318047	0	6.24E-18
45	55036	F	45	105860	0	2.50E-17
45	264530	F	45	264599	0	2.50E-17
48	179089	P	48	329264	-1	5.62E-17
54	30856	F	54	289930	-3	6.38E-17
51	37	P	51	45416	-2	6.99E-17
44	147443	P	44	164146	0	9.98E-17
44	291594	F	44	318384	0	9.98E-17
50	46115	F	50	302216	-2	2.69E-16
43	60592	F	43	192102	0	3.99E-16
43	158591	P	43	192102	0	3.99E-16
49	121362	F	49	123660	-2	1.03E-15
42	10898	P	42	53041	0	1.60E-15
42	54897	F	42	105722	0	1.60E-15
45	42491	P	45	278219	-1	3.37E-15

51	160006	F	51	177248	-3	3.43E-15
41	128557	P	41	323910	0	6.39E-15
41	190710	P	41	318525	0	6.39E-15
40	7009	P	40	94242	0	2.56E-14
40	217327	P	40	217327	0	2.56E-14
49	216024	F	49	304104	-3	4.85E-14
49	258321	P	49	309566	-3	4.85E-14
43	42490	P	43	192102	-1	5.15E-14
46	158592	P	46	278218	-2	5.81E-14
39	9999	P	39	154047	0	1.02E-13
39	74086	P	39	282982	0	1.02E-13
39	157492	F	39	178195	0	1.02E-13
48	46098	F	48	302199	-3	1.82E-13
48	160030	F	48	177272	-3	1.82E-13
48	160047	F	48	177289	-3	1.82E-13
42	11212	P	42	60592	-1	2.01E-13
42	11212	F	42	158592	-1	2.01E-13
42	11212	P	42	192102	-1	2.01E-13
45	258109	P	45	309784	-2	2.22E-13
38	57655	F	38	301886	0	4.09E-13
41	57501	F	41	252356	-1	7.86E-13
41	59021	P	41	190705	-1	7.86E-13
41	190705	P	41	274375	-1	7.86E-13
44	60590	F	44	278220	-2	8.50E-13
40	63387	P	40	265862	-1	3.07E-12
40	94981	F	40	292741	-1	3.07E-12
36	104734	F	36	273375	0	6.54E-12
36	153703	P	36	200427	0	6.54E-12
45	258973	P	45	308902	-3	9.56E-12
39	105406	F	39	282982	-1	1.20E-11
42	11212	F	42	42491	-2	1.24E-11
42	42491	F	42	214343	-2	1.24E-11
42	102849	P	42	248946	-2	1.24E-11
42	192102	F	42	278222	-2	1.24E-11
35	10903	F	35	324049	0	2.62E-11
35	42501	F	35	158602	0	2.62E-11
35	53043	P	35	324049	0	2.62E-11
35	53362	F	35	223038	0	2.62E-11
35	216956	F	35	237806	0	2.62E-11
44	45390	P	44	177802	-3	3.57E-11
44	45390	F	44	236163	-3	3.57E-11
38	11216	F	38	287192	-1	4.66E-11
38	42495	F	38	287192	-1	4.66E-11
38	46127	F	38	302228	-1	4.66E-11

38	178272	F	38	179125	-1	4.66E-11
34	34789	P	34	329234	0	1.05E-10
34	42501	P	34	60590	0	1.05E-10
34	64601	P	34	176806	0	1.05E-10
43	16647	F	43	111862	-3	1.33E-10
40	34644	P	40	293048	-2	1.79E-10
40	53357	F	40	255656	-2	1.79E-10
40	101287	P	40	293048	-2	1.79E-10
40	202920	P	40	216076	-2	1.79E-10
40	214345	F	40	287190	-2	1.79E-10
37	53645	F	37	255834	-1	1.82E-10
37	53645	F	37	317981	-1	1.82E-10
33	4771	F	33	246486	0	4.19E-10
33	34328	F	33	83810	0	4.19E-10
33	106161	F	33	262110	0	4.19E-10
33	181512	F	33	265873	0	4.19E-10
42	11212	P	42	278222	-3	4.95E-10
42	60592	P	42	214343	-3	4.95E-10
42	158592	F	42	214343	-3	4.95E-10
42	192102	P	42	214343	-3	4.95E-10
42	214343	P	42	278222	-3	4.95E-10
39	258331	P	39	309566	-2	6.82E-10
36	74123	P	36	179056	-1	7.07E-10
36	158602	P	36	278218	-1	7.07E-10
36	221953	P	36	295324	-1	7.07E-10
32	7556	F	32	293048	0	1.67E-09
32	42501	P	32	192102	0	1.67E-09
32	60600	F	32	104114	0	1.67E-09
32	61751	P	32	61751	0	1.67E-09
32	104114	P	32	158594	0	1.67E-09
32	104114	F	32	192110	0	1.67E-09
32	105412	P	32	203279	0	1.67E-09
32	153694	P	32	314956	0	1.67E-09
32	159533	P	32	289906	0	1.67E-09
32	188960	F	32	289617	0	1.67E-09
32	190673	F	32	238537	0	1.67E-09
32	193906	P	32	193906	0	1.67E-09
32	202238	F	32	257783	0	1.67E-09
38	60592	P	38	287192	-2	2.59E-09
38	158596	F	38	287192	-2	2.59E-09
38	192102	P	38	287192	-2	2.59E-09
38	278222	P	38	287192	-2	2.59E-09
35	39374	P	35	262358	-1	2.75E-09
35	211580	P	35	240425	-1	2.75E-09

35	216307	P	35	216307	-1	2.75E-09
31	50877	F	31	90952	0	6.7E-09
31	57511	P	31	178187	0	6.7E-09
31	73609	P	31	113162	0	6.7E-09
31	90915	F	31	216023	0	6.7E-09
31	100830	F	31	122772	0	6.7E-09
31	178187	P	31	252366	0	6.7E-09
40	11216	F	40	214347	-3	6.82E-09
37	34651	F	37	101294	-2	9.8E-09
37	79631	F	37	176941	-2	9.8E-09
37	122979	F	37	200426	-2	9.8E-09
37	122980	P	37	153702	-2	9.8E-09
37	160058	F	37	177300	-2	9.8E-09
34	56092	F	34	223592	-1	1.07E-08
34	67164	F	34	87384	-1	1.07E-08
34	104643	P	34	148273	-1	1.07E-08
34	122820	P	34	232533	-1	1.07E-08
34	203649	F	34	318656	-1	1.07E-08
39	34911	F	39	235875	-3	2.52E-08
39	104922	F	39	151282	-3	2.52E-08
30	12	P	30	45460	0	2.68E-08
30	12	F	30	177748	0	2.68E-08
30	12	P	30	236231	0	2.68E-08
30	22284	P	30	121632	0	2.68E-08
30	22284	P	30	240772	0	2.68E-08
30	42503	F	30	214355	0	2.68E-08
30	69981	P	30	204164	0	2.68E-08
30	105416	P	30	300881	0	2.68E-08
30	111222	F	30	127292	0	2.68E-08
30	163832	P	30	231213	0	2.68E-08
30	177904	F	30	269209	0	2.68E-08
30	190672	P	30	301898	0	2.68E-08
30	208540	P	30	225467	0	2.68E-08
30	236075	P	30	269209	0	2.68E-08
36	105406	F	36	153730	-2	3.71E-08
33	179268	P	33	180493	-1	4.15E-08
33	259509	P	33	284247	-1	4.15E-08
33	303070	P	33	330102	-1	4.15E-08
35	16655	F	35	111870	-2	1.4E-07
35	57530	P	35	157457	-2	1.4E-07
35	77639	P	35	240140	-2	1.4E-07
35	104923	F	35	259398	-2	1.4E-07
35	223038	F	35	255661	-2	1.4E-07
32	7556	P	32	34652	-1	1.61E-07

32	7556	P	32	101295	-1	1.61E-07
32	11133	F	32	203356	-1	1.61E-07
32	11214	P	32	104114	-1	1.61E-07
32	11222	F	32	42501	-1	1.61E-07
32	42493	P	32	104114	-1	1.61E-07
32	74087	F	32	203279	-1	1.61E-07
32	175520	P	32	269784	-1	1.61E-07
32	202928	P	32	216076	-1	1.61E-07
32	203279	P	32	282988	-1	1.61E-07
37	46077	F	37	302178	-3	3.43E-07
37	104922	P	37	284083	-3	3.43E-07
37	168098	F	37	293352	-3	3.43E-07
34	45400	P	34	177802	-2	5.29E-07
34	45400	F	34	236173	-2	5.29E-07
34	51005	F	34	211627	-2	5.29E-07
34	100700	P	34	100710	-2	5.29E-07
34	100700	F	34	100718	-2	5.29E-07
34	130470	P	34	161424	-2	5.29E-07
34	130706	F	34	184207	-2	5.29E-07
31	30731	P	31	161433	-1	6.23E-07
31	51400	F	31	71047	-1	6.23E-07
31	53173	F	31	57352	-1	6.23E-07
31	60599	F	31	256264	-1	6.23E-07
31	104733	P	31	238234	-1	6.23E-07
31	147220	P	31	245471	-1	6.23E-07
31	158596	P	31	256264	-1	6.23E-07
31	161396	F	31	176728	-1	6.23E-07
31	167151	F	31	307174	-1	6.23E-07
31	192109	F	31	256264	-1	6.23E-07
36	30726	F	36	130459	-3	1.26E-06
36	74089	P	36	153730	-3	1.26E-06
36	153730	F	36	282982	-3	1.26E-06
33	43054	P	33	197281	-2	1.99E-06
33	45698	P	33	221553	-2	1.99E-06
33	54260	F	33	271256	-2	1.99E-06
33	79641	F	33	176951	-2	1.99E-06
33	100714	R	33	100714	-2	1.99E-06
33	100714	C	33	100715	-2	1.99E-06
33	100715	R	33	100715	-2	1.99E-06
33	153163	F	33	248443	-2	1.99E-06
30	33115	F	30	44546	-1	2.41E-06
30	37502	P	30	255900	-1	2.41E-06
30	48957	F	30	268622	-1	2.41E-06
30	65271	F	30	235696	-1	2.41E-06

30	74085	F	30	300881	-1	2.41E-06
30	87926	F	30	91565	-1	2.41E-06
30	90916	F	30	304104	-1	2.41E-06
30	99149	F	30	244981	-1	2.41E-06
30	99467	F	30	151590	-1	2.41E-06
30	104114	F	30	256265	-1	2.41E-06
30	135618	F	30	235428	-1	2.41E-06
30	137965	P	30	231479	-1	2.41E-06
30	143425	F	30	178318	-1	2.41E-06
30	153736	P	30	203281	-1	2.41E-06
30	166957	P	30	248748	-1	2.41E-06
30	192959	P	30	301142	-1	2.41E-06
30	203277	F	30	300881	-1	2.41E-06
30	214355	P	30	278222	-1	2.41E-06
30	214355	F	30	287200	-1	2.41E-06
30	230717	P	30	315285	-1	2.41E-06
30	238233	P	30	273376	-1	2.41E-06
30	238536	P	30	301898	-1	2.41E-06
30	256482	P	30	311031	-1	2.41E-06
30	258988	P	30	308902	-1	2.41E-06
35	11207	F	35	214338	-3	4.62E-06
35	45584	P	35	177155	-3	4.62E-06
35	258770	P	35	309115	-3	4.62E-06
32	11222	P	32	278222	-2	7.48E-06
32	43188	P	32	252076	-2	7.48E-06
32	104114	F	32	278230	-2	7.48E-06
32	106701	F	32	213586	-2	7.48E-06
32	151283	F	32	259398	-2	7.48E-06
32	173028	F	32	225164	-2	7.48E-06
34	175486	P	34	269814	-3	0.0000169
31	37	F	31	177771	-2	0.000028
31	37	P	31	236207	-2	0.000028
31	67	F	31	177799	-2	0.000028
31	67	P	31	236179	-2	0.000028
31	11216	P	31	256264	-2	0.000028
31	24514	P	31	64090	-2	0.000028
31	42495	P	31	256264	-2	0.000028
31	53538	F	31	57957	-2	0.000028
31	54875	P	31	302981	-2	0.000028
31	75296	P	31	145074	-2	0.000028
31	94167	F	31	284779	-2	0.000028
31	105419	F	31	282995	-2	0.000028
31	105603	P	31	123731	-2	0.000028
31	123055	P	31	175781	-2	0.000028

31	130666	F	31	184172	-2	0.000028
31	143917	P	31	197963	-2	0.000028
31	161436	F	31	176761	-2	0.000028
31	237264	F	31	247195	-2	0.000028
31	282990	P	31	300882	-2	0.000028
33	50846	F	33	304111	-3	0.0000617
33	63906	P	33	279209	-3	0.0000617
33	243744	F	33	281279	-3	0.0000617
33	258309	P	33	309594	-3	0.0000617
30	10432	F	30	304071	-2	0.000105
30	11168	F	30	203080	-2	0.000105
30	11982	P	30	13091	-2	0.000105
30	28062	F	30	47127	-2	0.000105
30	30880	F	30	289954	-2	0.000105
30	34790	F	30	301895	-2	0.000105
30	42508	P	30	278217	-2	0.000105
30	63387	P	30	181511	-2	0.000105
30	67192	P	30	286064	-2	0.000105
30	70210	F	30	134219	-2	0.000105
30	75926	P	30	237334	-2	0.000105
30	85337	P	30	223609	-2	0.000105
30	93765	P	30	123809	-2	0.000105
30	95791	F	30	285432	-2	0.000105
30	99361	F	30	295122	-2	0.000105
30	104114	P	30	214347	-2	0.000105
30	104114	P	30	287192	-2	0.000105
30	104737	F	30	105712	-2	0.000105
30	105712	F	30	273378	-2	0.000105
30	111156	P	30	329306	-2	0.000105
30	146240	P	30	146240	-2	0.000105
30	148294	P	30	148294	-2	0.000105
30	176561	P	30	318840	-2	0.000105
30	179106	F	30	284254	-2	0.000105
30	208720	P	30	276436	-2	0.000105
30	246945	P	30	271842	-2	0.000105
30	301895	P	30	329237	-2	0.000105
30	306168	P	30	306168	-2	0.000105
32	11197	P	32	20670	-3	0.000224
32	123729	P	32	151221	-3	0.000224
32	130572	F	32	184068	-3	0.000224
32	139284	F	32	173002	-3	0.000224
32	181699	F	32	225331	-3	0.000224
32	214346	P	32	256264	-3	0.000224
32	256264	P	32	287191	-3	0.000224

32	283200	F	32	293442	-3	0.000224
31	18	F	31	153595	-3	0.000813
31	30730	P	31	176759	-3	0.000813
31	45601	P	31	177142	-3	0.000813
31	50845	F	31	216030	-3	0.000813
31	54953	F	31	167920	-3	0.000813
31	55426	F	31	273906	-3	0.000813
31	57499	P	31	203108	-3	0.000813
31	57664	P	31	238538	-3	0.000813
31	57667	P	31	190671	-3	0.000813
31	77643	P	31	319382	-3	0.000813
31	83842	P	31	190672	-3	0.000813
31	83843	F	31	301898	-3	0.000813
31	86247	F	31	209341	-3	0.000813
31	87055	F	31	103658	-3	0.000813
31	91046	F	31	273592	-3	0.000813
31	96173	F	31	228147	-3	0.000813
31	121808	F	31	284344	-3	0.000813
31	130430	P	31	184468	-3	0.000813
31	153738	P	31	300882	-3	0.000813
31	157456	P	31	203105	-3	0.000813
31	168500	F	31	236671	-3	0.000813
31	197670	P	31	226803	-3	0.000813
31	238648	F	31	248017	-3	0.000813
31	256076	P	31	311327	-3	0.000813
31	256264	F	31	278229	-3	0.000813
31	303338	F	31	303395	-3	0.000813
30	562	F	30	179821	-3	0.00294
30	11450	P	30	39777	-3	0.00294
30	16659	P	30	79403	-3	0.00294
30	30726	F	30	269846	-3	0.00294
30	34790	F	30	57664	-3	0.00294
30	35478	P	30	123124	-3	0.00294
30	45341	F	30	46546	-3	0.00294
30	50318	P	30	156499	-3	0.00294
30	50559	P	30	156683	-3	0.00294
30	54887	F	30	104737	-3	0.00294
30	54887	F	30	273378	-3	0.00294
30	55067	P	30	120566	-3	0.00294
30	57664	P	30	329237	-3	0.00294
30	66939	P	30	283141	-3	0.00294
30	69063	P	30	301108	-3	0.00294
30	77649	P	30	240135	-3	0.00294
30	83842	P	30	238537	-3	0.00294

30	90949	P	30	167787	-3	0.00294
30	90951	P	30	248939	-3	0.00294
30	97340	F	30	213318	-3	0.00294
30	100710	F	30	100728	-3	0.00294
30	118253	F	30	296560	-3	0.00294
30	134048	P	30	266702	-3	0.00294
30	203108	P	30	252355	-3	0.00294
30	214227	P	30	239778	-3	0.00294
30	256813	P	30	310730	-3	0.00294
30	258128	P	30	309780	-3	0.00294
30	258815	P	30	309075	-3	0.00294
30	284254	P	30	329265	-3	0.00294

Table S5 The homologous DNA fragment in the bitter gourd mitochondrial genome.

Number	% Identity	Alignment Length	Mismatches	Gap Openings	Alignment start (chloroplast genome)	Alignment end (chloroplast genome)	Alignment start (mitochondrial genome)	Alignment end (mitochondrial genome)	E-value	Bit Score
1	94.609	7382	222	64	143920	151174	141461	134129	0	11265
2	94.609	7382	222	64	95519	102773	134129	141461	0	11265
3	87.103	1070	99	14	67201	68257	9.37E+04	92682	0.00E+00	1175
4	97.743	576	11	1	21623	22198	3.23E+05	322901	0.00E+00	990
5	88.588	701	59	10	18840	19536	6.30E+04	62299	0.00E+00	832
6	90.805	435	24	6	18144	18578	6.34E+04	62969	5.40E-161	568
7	83.333	492	46	21	31380	31850	2.26E+05	225802	4.45E-117	422
8	80	440	61	15	69469	69889	9.21E+04	91625	2.17E-80	300
9	89.017	173	17	2	69972	70142	9.16E+04	91389	2.91E-54	213
10	95.238	84	3	1	112752	112834	167186	167103	8.4E-30	132
11	95.238	84	3	1	133859	133941	167103	167186	8.4E-30	132
12	93.258	89	4	2	161787	161873	122171	122083	3.02E-29	130
13	93.258	89	4	2	84820	84906	122083	122171	3.02E-29	130
14	93.671	79	5	0	55512	55590	156712	156634	6.53E-26	119
15	100	49	0	0	142471	142519	83922	83874	1.42E-17	91.6
16	100	49	0	0	104174	104222	83874	83922	1.42E-17	91.6
17	97.5	40	1	0	124218	124257	141244	141283	6.67E-11	69.4
18	97.436	39	1	0	11898	11936	101646	101684	2.40E-10	67.6
19	81.609	87	4	6	90051	90125	31717	31631	1.12E-08	62.1
20	81.609	87	4	6	156568	156642	31631	31717	1.12E-08	62.1

Table S6 The RNA editing events prediction in bitter gourd

Gene	Base	Aa	Triplet pos.	Bases	Codon	Aa change
atp1	302	101	2	C→U	GCG→GUG	A→V
atp1	488	163	2	C→U	GCG→GUG	A→V
atp1	514	172	1	C→U	CGU→UGU	R→C
atp1	752	251	2	C→U	GCU→GUU	A→V
atp1	799	267	1	C→U	CCU→UCU	P→S
atp1	1108	370	1	C→U	CCU→UCU	P→S
atp1	1247	416	2	C→U	UCA→UUA	S→L
atp1	1316	439	2	C→U	GCG→GUG	A→V
atp4	59	20	2	C→U	UCU→UUU	S→F
atp4	89	30	2	C→U	UCA→UUA	S→L
atp4	176	59	2	C→U	GCG→GUG	A→V
atp4	215	72	2	C→U	UCG→UUG	S→L
atp4	227	76	2	C→U	CCC→CUC	P→L
atp4	248	83	2	C→U	CCU→CUU	P→L
atp4	251	84	2	C→U	CCG→CUG	P→L
atp4	395	132	2	C→U	UCA→UUA	S→L
atp4	407	136	2	C→U	CCA→CUA	P→L
atp4	416	139	2	C→U	ACU→AUU	T→I
atp6	119	40	2	C→U	ACU→AUU	T→I
atp6	329	110	2	C→U	CCC→CUC	P→L
atp6	373	125	1	C→U	CUC→UUC	L→F
atp6	461	154	2	C→U	UCA→UUA	S→L
atp6	520	174	1	C→U	CCU→UCU	P→S
atp6	523	175	1	C→U	CAU→UAU	H→Y
atp6	545	182	2	C→U	UCA→UUA	S→L
atp6	587	196	2	C→U	UCA→UUA	S→L
atp6	608	203	2	C→U	UCC→UUC	S→F
atp6	722	241	2	C→U	UCA→UUA	S→L
atp6	730	244	1	C→U	CAU→UAU	H→Y
atp6	737	246	2	C→U	UCU→UUU	S→F
atp6	746	249	2	C→U	UCA→UUA	S→L
atp8	47	16	2	C→U	UCA→UUA	S→L
atp8	76	26	1	C→U	CCC→UCC	P→S
atp9	50	17	2	C→U	UCA→UUA	S→L
atp9	59	20	2	C→U	GCC→GUC	A→V
atp9	200	67	2	C→U	GCC→GUC	A→V
ccmB	28	10	1	C→U	CAU→UAU	H→Y
ccmB	71	24	2	C→U	CCA→CUA	P→L
ccmB	80	27	2	C→U	UCG→UUG	S→L
ccmB	128	43	2	C→U	UCA→UUA	S→L
ccmB	133	45	1	C→U	CAU→UAU	H→Y

ccmB	137	46	2	C→U	UCC→UUC	S→F
ccmB	149	50	2	C→U	CCG→CUG	P→L
ccmB	154	52	1	C→U	CGG→UGG	R→W
ccmB	160	54	1	C→U	CCU→UCU	P→S
ccmB	164	55	2	C→U	CCG→CUG	P→L
ccmB	172	58	1	C→U	CCU→UCU	P→S
ccmB	179	60	2	C→U	CCU→CUU	P→L
ccmB	181	61	1	C→U	CCC→UCC	P→S
ccmB	193	65	1	C→U	CCU→UCU	P→S
ccmB	194	65	2	C→U	UCU→UUU	S→F
ccmB	286	96	1	C→U	CGG→UGG	R→W
ccmB	338	113	2	C→U	CCG→CUG	P→L
ccmB	367	123	1	C→U	CGG→UGG	R→W
ccmB	424	142	1	C→U	CGU→UGU	R→C
ccmB	428	143	2	C→U	UCG→UUG	S→L
ccmB	467	156	2	C→U	UCG→UUG	S→L
ccmB	476	159	2	C→U	CCA→CUA	P→L
ccmB	485	162	2	C→U	UCA→UUA	S→L
ccmB	494	165	2	C→U	UCA→UUA	S→L
ccmB	503	168	2	C→U	CCA→CUA	P→L
ccmB	506	169	2	C→U	ACC→AUC	T→I
ccmB	512	171	2	C→U	UCU→UUU	S→F
ccmB	514	172	1	C→U	CGU→UGU	R→C
ccmB	551	184	2	C→U	UCA→UUA	S→L
ccmB	554	185	2	C→U	UCG→UUG	S→L
ccmB	566	189	2	C→U	UCC→UUC	S→F
ccmB	569	190	2	C→U	UCC→UUC	S→F
ccmB	572	191	2	C→U	CCG→CUG	P→L
ccmB	596	199	2	C→U	UCG→UUG	S→L
ccmC	103	35	1	C→U	CAU→UAU	H→Y
ccmC	115	39	1	C→U	CGG→UGG	R→W
ccmC	133	45	1	C→U	CUU→UUU	L→F
ccmC	179	60	2	C→U	GCG→GUG	A→V
ccmC	184	62	1	C→U	CGG→UGG	R→W
ccmC	266	89	2	C→U	UCU→UUU	S→F
ccmC	281	94	2	C→U	ACA→AUA	T→I
ccmC	331	111	1	C→U	CGG→UGG	R→W
ccmC	395	132	2	C→U	UCG→UUG	S→L
ccmC	400	134	1	C→U	CUU→UUU	L→F
ccmC	421	141	1	C→U	CGU→UGU	R→C
ccmC	436	146	1	C→U	CCU→UCU	P→S
ccmC	446	149	2	C→U	CCG→CUG	P→L
ccmC	458	153	2	C→U	UCA→UUA	S→L
ccmC	463	155	1	C→U	CGU→UGU	R→C

ccmC	473	158	2	C→U	CCG→CUG	P→L
ccmC	521	174	2	C→U	UCG→UUG	S→L
ccmC	548	183	2	C→U	UCU→UUU	S→F
ccmC	568	190	1	C→U	CCU→UCU	P→S
ccmC	575	192	2	C→U	CCC→CUC	P→L
ccmC	608	203	2	C→U	CCC→CUC	P→L
ccmC	614	205	2	C→U	UCA→UUA	S→L
ccmC	619	207	1	C→U	CGU→UGU	R→C
ccmC	650	217	2	C→U	CCU→CUU	P→L
ccmC	656	219	2	C→U	CCA→CUA	P→L
ccmC	665	222	2	C→U	CCC→CUC	P→L
ccmC	673	225	1	C→U	CCU→UCU	P→S
ccmFC	38	13	2	C→U	UCC→UUC	S→F
ccmFC	50	17	2	C→U	CCU→CUU	P→L
ccmFC	52	18	1	C→U	CGU→UGU	R→C
ccmFC	100	34	1	C→U	CCC→UCC	P→S
ccmFC	104	35	2	C→U	ACA→AUA	T→I
ccmFC	116	39	2	C→U	UCU→UUU	S→F
ccmFC	119	40	2	C→U	UCC→UUC	S→F
ccmFC	143	48	2	C→U	CCU→CUU	P→L
ccmFC	157	53	1	C→U	CCU→UCU	P→S
ccmFC	160	54	1	C→U	CUU→UUU	L→F
ccmFC	293	98	2	C→U	UCA→UUA	S→L
ccmFC	298	100	1	C→U	CGU→UGU	R→C
ccmFC	322	108	1	C→U	CUU→UUU	L→F
ccmFC	335	112	2	C→U	UCC→UUC	S→F
ccmFC	394	132	1	C→U	CGU→UGU	R→C
ccmFC	400	134	1	C→U	CUC→UUC	L→F
ccmFC	407	136	2	C→U	UCU→UUU	S→F
ccmFC	587	196	2	C→U	UCG→UUG	S→L
ccmFC	701	234	2	C→U	CCA→CUA	P→L
ccmFC	872	291	2	C→U	UCU→UUU	S→F
ccmFC	925	309	1	C→U	CGG→UGG	R→W
ccmFC	1012	338	1	C→U	CAU→UAU	H→Y
ccmFC	1133	378	2	C→U	CCA→CUA	P→L
ccmFC	1172	391	2	C→U	UCC→UUC	S→F
ccmFC	1228	410	1	C→U	CGG→UGG	R→W
ccmFC	1262	421	2	C→U	UCG→UUG	S→L
ccmFN	29	10	2	C→U	UCG→UUG	S→L
ccmFN	37	13	1	C→U	CCC→UCC	P→S
ccmFN	98	33	2	C→U	CCU→CUU	P→L
ccmFN	137	46	2	C→U	UCG→UUG	S→L
ccmFN	142	48	1	C→U	CGU→UGU	R→C
ccmFN	151	51	1	C→U	CCU→UCU	P→S

ccmFN	248	83	2	C→U	UCA→UUA	S→L
ccmFN	256	86	1	C→U	CGG→UGG	R→W
ccmFN	263	88	2	C→U	CCA→CUA	P→L
ccmFN	356	119	2	C→U	UCC→UUC	S→F
ccmFN	365	122	2	C→U	UCG→UUG	S→L
ccmFN	382	128	1	C→U	CCC→UCC	P→S
ccmFN	544	182	1	C→U	CCU→UCU	P→S
ccmFN	548	183	2	C→U	CCG→CUG	P→L
ccmFN	688	230	1	C→U	CCU→UCU	P→S
ccmFN	689	230	2	C→U	UCU→UUU	S→F
ccmFN	736	246	1	C→U	CGU→UGU	R→C
ccmFN	758	253	2	C→U	UCA→UUA	S→L
ccmFN	770	257	2	C→U	CCA→CUA	P→L
ccmFN	785	262	2	C→U	UCA→UUA	S→L
ccmFN	934	312	1	C→U	CGC→UGC	R→C
ccmFN	1178	393	2	C→U	UCG→UUG	S→L
ccmFN	1261	421	1	C→U	CGG→UGG	R→W
ccmFN	1289	430	2	C→U	CCA→CUA	P→L
ccmFN	1306	436	1	C→U	CAU→UAU	H→Y
ccmFN	1321	441	1	C→U	CGG→UGG	R→W
ccmFN	1339	447	1	C→U	CGG→UGG	R→W
ccmFN	1372	458	1	C→U	CGG→UGG	R→W
ccmFN	1390	464	1	C→U	CGU→UGU	R→C
ccmFN	1414	472	1	C→U	CUU→UUU	L→F
ccmFN	1433	478	2	C→U	UCG→UUG	S→L
ccmFN	1457	486	2	C→U	CCA→CUA	P→L
ccmFN	1469	490	2	C→U	UCA→UUA	S→L
ccmFN	1481	494	2	C→U	UCA→UUA	S→L
ccmFN	1504	502	1	C→U	CCC→UCC	P→S
ccmFN	1523	508	2	C→U	ACA→AUA	T→I
ccmFN	1682	561	2	C→U	UCG→UUG	S→L
ccmFN	1693	565	1	C→U	CAA→UAA	Q→*
cox1	92	31	2	C→U	ACU→AUU	T→I
cox1	134	45	2	C→U	ACA→AUA	T→I
cox1	199	67	1	C→U	CAU→UAU	H→Y
cox1	205	69	1	C→U	CUU→UUU	L→F
cox1	281	94	2	C→U	UCU→UUU	S→F
cox1	293	98	2	C→U	UCU→UUU	S→F
cox1	485	162	2	C→U	GCA→GUA	A→V
cox1	491	164	2	C→U	UCU→UUU	S→F
cox1	496	166	1	C→U	CUU→UUU	L→F
cox1	554	185	2	C→U	UCC→UUC	S→F
cox1	590	197	2	C→U	UCA→UUA	S→L
cox1	629	210	2	C→U	CCA→CUA	P→L

cox1	754	252	1	C→U	CGG→UGG	R→W
cox1	785	262	2	C→U	CCC→CUC	P→L
cox1	800	267	2	C→U	UCC→UUC	S→F
cox1	1076	359	2	C→U	UCC→UUC	S→F
cox1	1118	373	2	C→U	CCG→CUG	P→L
cox1	1225	409	1	C→U	CAC→UAC	H→Y
cox1	1313	438	2	C→U	CCG→CUG	P→L
cox1	1318	440	1	C→U	CUC→UUC	L→F
cox2	203	68	2	C→U	ACU→AUU	T→I
cox2	229	77	1	C→U	CCU→UCU	P→S
cox2	260	87	2	C→U	CCA→CUA	P→L
cox2	326	109	2	C→U	GCU→GUU	A→V
cox2	346	116	1	C→U	CGG→UGG	R→W
cox2	521	174	2	C→U	GCC→GUC	A→V
cox2	535	179	1	C→U	CGU→UGU	R→C
cox2	550	184	1	C→U	CCU→UCU	P→S
cox2	704	235	2	C→U	ACG→AUG	T→M
cox3	262	88	1	C→U	CAC→UAC	H→Y
cox3	266	89	2	C→U	UCA→UUA	S→L
cox3	301	101	1	C→U	CUC→UUC	L→F
cox3	385	129	1	C→U	CAU→UAU	H→Y
cox3	454	152	1	C→U	CUU→UUU	L→F
cox3	469	157	1	C→U	CGG→UGG	R→W
cox3	473	158	2	C→U	GCU→GUU	A→V
cox3	524	175	2	C→U	CCA→CUA	P→L
cox3	547	183	1	C→U	CCU→UCU	P→S
cox3	548	183	2	C→U	UCU→UUU	S→F
cox3	613	205	1	C→U	CAU→UAU	H→Y
cox3	616	206	1	C→U	CAU→UAU	H→Y
cox3	916	306	1	C→U	CGG→UGG	R→W
cob	37	13	1	C→U	CCU→UCU	P→S
cob	38	13	2	C→U	UCU→UUU	S→F
cob	115	39	1	C→U	CCG→UCG	P→S
cob	283	95	1	C→U	CUC→UUC	L→F
cob	295	99	1	C→U	CAC→UAC	H→Y
cob	355	119	1	C→U	CGG→UGG	R→W
cob	473	158	2	C→U	GCC→GUC	A→V
cob	853	285	1	C→U	CAU→UAU	H→Y
cob	875	292	2	C→U	CCU→CUU	P→L
cob	908	303	2	C→U	CCA→CUA	P→L
cob	1045	349	1	C→U	CCU→UCU	P→S
cob	1046	349	2	C→U	UCU→UUU	S→F
cob	1126	376	1	C→U	CCG→UCG	P→S
matR	85	29	1	C→U	CGC→UGC	R→C

matR	166	56	1	C→U	CAC→UAC	H→Y
matR	236	79	2	C→U	UCC→UUC	S→F
matR	317	106	2	C→U	UCG→UUG	S→L
matR	326	109	2	C→U	CCA→CUA	P→L
matR	413	138	2	C→U	UCG→UUG	S→L
matR	481	161	1	C→U	CCC→UCC	P→S
matR	482	161	2	C→U	UCC→UUC	S→F
matR	1040	347	2	C→U	CCU→CUU	P→L
matR	1385	462	2	C→U	GCG→GUG	A→V
matR	1519	507	1	C→U	CCC→UCC	P→S
matR	1655	552	2	C→U	UCC→UUC	S→F
matR	1732	578	1	C→U	CAC→UAC	H→Y
matR	1820	607	2	C→U	CCA→CUA	P→L
mttB	20	7	2	C→U	UCC→UUC	S→F
mttB	41	14	2	C→U	CCG→CUG	P→L
mttB	47	16	2	C→U	ACU→AUU	T→I
mttB	79	27	1	C→U	CGG→UGG	R→W
mttB	94	32	1	C→U	CUU→UUU	L→F
mttB	127	43	1	C→U	CCG→UCG	P→S
mttB	143	48	2	C→U	UCU→UUU	S→F
mttB	146	49	2	C→U	CCA→CUA	P→L
mttB	203	68	2	C→U	UCA→UUA	S→L
mttB	217	73	1	C→U	CCG→UCG	P→S
mttB	239	80	2	C→U	UCA→UUA	S→L
mttB	251	84	2	C→U	UCU→UUU	S→F
mttB	277	93	1	C→U	CAU→UAU	H→Y
mttB	343	115	1	C→U	CUC→UUC	L→F
mttB	359	120	2	C→U	UCU→UUU	S→F
mttB	361	121	1	C→U	CGC→UGC	R→C
mttB	368	123	2	C→U	UCC→UUC	S→F
mttB	388	130	1	C→U	CUU→UUU	L→F
mttB	391	131	1	C→U	CCC→UCC	P→S
mttB	394	132	1	C→U	CGG→UGG	R→W
mttB	422	141	2	C→U	CCA→CUA	P→L
mttB	452	151	2	C→U	UCG→UUG	S→L
mttB	487	163	1	C→U	CAU→UAU	H→Y
mttB	512	171	2	C→U	UCG→UUG	S→L
mttB	556	186	1	C→U	CGU→UGU	R→C
mttB	563	188	2	C→U	CCA→CUA	P→L
mttB	625	209	1	C→U	CCG→UCG	P→S
mttB	647	216	2	C→U	UCC→UUC	S→F
mttB	682	228	1	C→U	CGU→UGU	R→C
mttB	728	243	2	C→U	UCG→UUG	S→L
nad1	208	70	1	C→U	CCA→UCA	P→S

nad1	299	100	2	C→U	UCG→UUG	S→L
nad1	430	144	1	C→U	CGG→UGG	R→W
nad1	467	156	2	C→U	UCA→UUA	S→L
nad1	601	201	1	C→U	CCU→UCU	P→S
nad1	635	212	2	C→U	ACU→AUU	T→I
nad1	803	268	2	C→U	UCA→UUA	S→L
nad1	842	281	2	C→U	UCU→UUU	S→F
nad1	847	283	1	C→U	CUU→UUU	L→F
nad1	904	302	1	C→U	CUC→UUC	L→F
nad1	938	313	2	C→U	CCG→CUG	P→L
nad1	1045	349	1	C→U	CGG→UGG	R→W
nad2	347	116	2	C→U	ACA→AUA	T→I
nad2	482	161	2	C→U	UCA→UUA	S→L
nad2	578	193	2	C→U	UCG→UUG	S→L
nad2	737	246	2	C→U	UCU→UUU	S→F
nad2	770	257	2	C→U	GCA→GUA	A→V
nad2	793	265	1	C→U	CCU→UCU	P→S
nad2	815	272	2	C→U	CCC→CUC	P→L
nad2	818	273	2	C→U	ACC→AUC	T→I
nad2	1043	348	2	C→U	ACA→AUA	T→I
nad2	1100	367	2	C→U	UCA→UUA	S→L
nad2	1169	390	2	C→U	ACC→AUC	T→I
nad2	1310	437	2	C→U	GCC→GUC	A→V
nad3	167	56	2	C→U	UCC→UUC	S→F
nad3	229	77	1	C→U	CUU→UUU	L→F
nad3	245	82	2	C→U	ACC→AUC	T→I
nad3	251	84	2	C→U	UCC→UUC	S→F
nad3	271	91	1	C→U	CUC→UUC	L→F
nad3	296	99	2	C→U	UCU→UUU	S→F
nad3	340	114	1	C→U	CUC→UUC	L→F
nad4	212	71	2	C→U	CCU→CUU	P→L
nad4	328	110	1	C→U	CCU→UCU	P→S
nad4	329	110	2	C→U	UCU→UUU	S→F
nad4	349	117	1	C→U	CCC→UCC	P→S
nad4	350	117	2	C→U	UCC→UUC	S→F
nad4	362	121	2	C→U	CCA→CUA	P→L
nad4	436	146	1	C→U	CUU→UUU	L→F
nad4	487	163	1	C→U	CUU→UUU	L→F
nad4	497	166	2	C→U	ACA→AUA	T→I
nad4	521	174	2	C→U	UCA→UUA	S→L
nad4	613	205	1	C→U	CAU→UAU	H→Y
nad4	697	233	1	C→U	CAC→UAC	H→Y
nad4	716	239	2	C→U	UCA→UUA	S→L
nad4	749	250	2	C→U	UCC→UUC	S→F

nad4	872	291	2	C→U	ACU→AUU	T→I
nad4	923	308	2	C→U	CCG→CUG	P→L
nad4	1055	352	2	C→U	UCC→UUC	S→F
nad4	1061	354	2	C→U	UCU→UUU	S→F
nad4	1067	356	2	C→U	ACU→AUU	T→I
nad4	1118	373	2	C→U	CCC→CUC	P→L
nad4	1160	387	2	C→U	ACA→AUA	T→I
nad4	1229	410	2	C→U	UCU→UUU	S→F
nad4	1268	423	2	C→U	CCA→CUA	P→L
nad4	1346	449	2	C→U	CCG→CUG	P→L
nad4L	35	12	2	C→U	UCU→UUU	S→F
nad4L	89	30	2	C→U	UCA→UUA	S→L
nad4L	104	35	2	C→U	UCA→UUA	S→L
nad4L	125	42	2	C→U	UCG→UUG	S→L
nad4L	152	51	2	C→U	UCG→UUG	S→L
nad4L	182	61	2	C→U	UCA→UUA	S→L
nad4L	191	64	2	C→U	CCA→CUA	P→L
nad5	116	39	2	C→U	UCG→UUG	S→L
nad5	212	71	2	C→U	GCU→GUU	A→V
nad5	239	80	2	C→U	CCG→CUG	P→L
nad5	395	132	2	C→U	UCU→UUU	S→F
nad5	502	168	1	C→U	CCU→UCU	P→S
nad5	503	168	2	C→U	UCU→UUU	S→F
nad5	611	204	2	C→U	GCC→GUC	A→V
nad5	614	205	2	C→U	CCC→CUC	P→L
nad5	649	217	1	C→U	CUU→UUU	L→F
nad5	689	230	2	C→U	UCG→UUG	S→L
nad5	695	232	2	C→U	ACU→AUU	T→I
nad5	701	234	2	C→U	UCA→UUA	S→L
nad5	740	247	2	C→U	UCG→UUG	S→L
nad5	836	279	2	C→U	ACU→AUU	T→I
nad5	1366	456	1	C→U	CAU→UAU	H→Y
nad5	1375	459	1	C→U	CCC→UCC	P→S
nad5	1397	466	2	C→U	UCA→UUA	S→L
nad5	1523	508	2	C→U	UCC→UUC	S→F
nad5	1535	512	2	C→U	GCU→GUU	A→V
nad5	1586	529	2	C→U	ACU→AUU	T→I
nad5	1619	540	2	C→U	CCC→CUC	P→L
nad6	70	24	1	C→U	CAU→UAU	H→Y
nad6	86	29	2	C→U	CCC→CUC	P→L
nad6	92	31	2	C→U	CCA→CUA	P→L
nad6	158	53	2	C→U	CCA→CUA	P→L
nad6	166	56	1	C→U	CAU→UAU	H→Y
nad7	38	13	2	C→U	UCG→UUG	S→L

nad7	44	15	2	C→U	UCC→UUC	S→F
nad7	77	26	2	C→U	UCA→UUA	S→L
nad7	83	28	2	C→U	UCA→UUA	S→L
nad7	200	67	2	C→U	UCU→UUU	S→F
nad7	209	70	2	C→U	UCA→UUA	S→L
nad7	224	75	2	C→U	ACG→AUG	T→M
nad7	244	82	1	C→U	CAU→UAU	H→Y
nad7	251	84	2	C→U	UCA→UUA	S→L
nad7	316	106	1	C→U	CGU→UGU	R→C
nad7	335	112	2	C→U	UCA→UUA	S→L
nad7	344	115	2	C→U	UCA→UUA	S→L
nad7	383	128	2	C→U	UCA→UUA	S→L
nad7	578	193	2	C→U	UCA→UUA	S→L
nad7	724	242	1	C→U	CAU→UAU	H→Y
nad7	739	247	1	C→U	CUU→UUU	L→F
nad7	769	257	1	C→U	CGC→UGC	R→C
nad7	926	309	2	C→U	UCA→UUA	S→L
nad7	1057	353	1	C→U	CGU→UGU	R→C
nad7	1079	360	2	C→U	UCU→UUU	S→F
nad7	1088	363	2	C→U	UCA→UUA	S→L
nad7	1103	368	2	C→U	UCU→UUU	S→F
nad7	1124	375	2	C→U	CCA→CUA	P→L
nad9	40	14	1	C→U	CUC→UUC	L→F
nad9	167	56	2	C→U	UCG→UUG	S→L
nad9	298	100	1	C→U	CCG→UCG	P→S
nad9	328	110	1	C→U	CGG→UGG	R→W
nad9	368	123	2	C→U	UCC→UUC	S→F
nad9	398	133	2	C→U	UCA→UUA	S→L
nad9	439	147	1	C→U	CUU→UUU	L→F
rpl2	206	69	2	C→U	CCA→CUA	P→L
rpl2	338	113	2	C→U	CCG→CUG	P→L
rpl2	731	244	2	C→U	GCU→GUU	A→V
rpl2	802	268	1	C→U	CUU→UUU	L→F
rpl2	1271	424	2	C→U	GCG→GUG	A→V
rpl5	35	12	2	C→U	UCA→UUA	S→L
rpl5	47	16	2	C→U	CCG→CUG	P→L
rpl5	92	31	2	C→U	UCG→UUG	S→L
rpl5	167	56	2	C→U	CCG→CUG	P→L
rpl5	169	57	1	C→U	CGC→UGC	R→C
rpl5	317	106	2	C→U	UCG→UUG	S→L
rpl5	329	110	2	C→U	UCG→UUG	S→L
rpl5	512	171	2	C→U	CCA→CUA	P→L
rpl5	515	172	2	C→U	CCG→CUG	P→L
rpl10	224	75	2	C→U	UCA→UUA	S→L

rpl10	319	107	1	C→U	CAU→UAU	H→Y
rpl16	37	13	1	C→U	CGU→UGU	R→C
rpl16	104	35	2	C→U	ACU→AUU	T→I
rpl16	208	70	1	C→U	CUC→UUC	L→F
rpl16	311	104	2	C→U	CCA→CUA	P→L
rpl16	377	126	2	C→U	CCA→CUA	P→L
rpl16	383	128	2	C→U	UCG→UUG	S→L
rps1	158	53	2	C→U	CCU→CUU	P→L
rps1	209	70	2	C→U	UCC→UUC	S→F
rps1	338	113	2	C→U	UCC→UUC	S→F
rps1	524	175	2	C→U	CCU→CUU	P→L
rps1	526	176	1	C→U	CUC→UUC	L→F
rps1	560	187	2	C→U	ACC→AUC	T→I
rps3	1481	494	2	C→U	UCA→UUA	S→L
rps4	49	17	1	C→U	CGG→UGG	R→W
rps4	133	45	1	C→U	CCG→UCG	P→S
rps4	164	55	2	C→U	UCA→UUA	S→L
rps4	184	62	1	C→U	CCC→UCC	P→S
rps4	193	65	1	C→U	CAU→UAU	H→Y
rps4	257	86	2	C→U	CCA→CUA	P→L
rps4	266	89	2	C→U	CCA→CUA	P→L
rps4	278	93	2	C→U	UCG→UUG	S→L
rps4	290	97	2	C→U	CCG→CUG	P→L
rps4	335	112	2	C→U	CCG→CUG	P→L
rps4	464	155	2	C→U	UCU→UUU	S→F
rps4	482	161	2	C→U	UCA→UUA	S→L
rps4	721	241	1	C→U	CAU→UAU	H→Y
rps4	775	259	1	C→U	CCU→UCU	P→S
rps4	905	302	2	C→U	UCG→UUG	S→L
rps4	916	306	1	C→U	CAU→UAU	H→Y
rps4	941	314	2	C→U	UCU→UUU	S→F
rps7	116	39	2	C→U	CCA→CUA	P→L
rps7	277	93	1	C→U	CUU→UUU	L→F
rps7	332	111	2	C→U	UCA→UUA	S→L
rps10	235	79	1	C→U	CGG→UGG	R→W
rps10	313	105	1	C→U	CUC→UUC	L→F
rps12	71	24	2	C→U	UCG→UUG	S→L
rps12	100	34	1	C→U	CGC→UGC	R→C
rps12	104	35	2	C→U	CCG→CUG	P→L
rps12	146	49	2	C→U	CCA→CUA	P→L
rps12	196	66	1	C→U	CAC→UAC	H→Y
rps12	269	90	2	C→U	UCG→UUG	S→L
rps12	284	95	2	C→U	UCC→UUC	S→F
rps13	319	107	1	C→U	CAU→UAU	H→Y

rps14	188	63	2	C→U	UCC→UUC	S→F
rps14	199	67	1	C→U	CCU→UCU	P→S
rps19	164	55	2	C→U	UCU→UUU	S→F
rps19	242	81	2	C→U	UCG→UUG	S→L
sdh3	67	23	1	C→U	CCA→UCA	P→S
sdh3	74	25	2	C→U	UCC→UUC	S→F
sdh3	127	43	1	C→U	CUU→UUU	L→F
sdh3	191	64	2	C→U	UCA→UUA	S→L
sdh3	199	67	1	C→U	CUC→UUC	L→F
sdh4	916	306	1	C→U	CGG→UGG	R→W

Table S7. Species information for RNA editing event reference

Accession number	Species
NC 026286.1	Acrasis kona
KF754799.1	Amborella trichopoda
NC 001284.2	Arabidopsis thaliana
NC 002511.2	Beta vulgaris subsp. vulgaris
NC 008285.1	Brassica napus
NC 004118.1	Chaetosphaeridium globosum
NC 005255.1	Chara vulgaris
NC 014043.1	Citrullus lanatus
NC 031696.1	Cocos nucifera
NC 014050.1	Cucurbita pepo
F010859.1	Isoetes engelmannii
KC821969.1	Liriodendron tulipifera
NC 016743.2	Lotus japonicus
NC 001660.1	Marchantia paleacea
NC 016742.1	Millettia pinnata
NC 002573.1	Naegleria gruberi
BA0000421	Nicotiana tabacum
NC 030900.1	Ophioglossum californicum
NC 011033.1	Oryza sativa Japonica Group
NC 007945.1	Physcomitrella patens
KX171638.1	Psilotum nudum
NC 0018231	Reclinomonas americana
JF338143.1	Selaginella moellendorffi
NC 014487.1	Silene latifolia
NC 012119.1	Vitis vinifera
